# Phytoacoustics: sound perception, mechanosensing, and biological responses to sound in plants

**DOI:** 10.1080/15592324.2026.2709223

**Published:** 2026-07-30

**Authors:** Michal Mos, Zuzanna E. Kassner, Paul R.H. Robson

**Affiliations:** a Plantcustic Limited, Glasgow, Scotland, UK; b IBERS, Aberystwyth University, University of Aberystwyth, Aberystwyth, Wales, UK

**Keywords:** Phytoacoustic, sound, physiology, signaling networks, metabolism, sensing mechanisms

## Abstract

Sound and vibration are emerging as biologically relevant mechanical inputs in plant systems. Plants are exposed to complex soundscapes generated by herbivores, pollinators, abiotic forces, and anthropogenic noise, and growing evidence shows that such stimuli can alter defense, pollination-related traits, drought tolerance, ripening, germination, growth and metabolite accumulation. In this review, we synthesize current knowledge on how plants perceive and respond to sound-related mechanical stimuli across ecological, physiological, and molecular scales. We focus on the strongest mechanistic candidates, including plasma membrane deformation, mechanosensitive ion channels, calcium influx, reactive oxygen species signaling, transcriptional reprogramming, and phytohormonal shifts. The available evidence indicates that plant responses to sound are highly context-dependent and shaped by stimulus frequency, intensity, temporal structure, delivery route, species identity, and developmental stage. We also highlight major unresolved questions, particularly concerning the primary sites of perception, the distinction between sound and other forms of mechanostimulation, and the need for stronger methodological standardization. Together, these findings support phytoacoustics as a promising framework for understanding sound as an environmental factor in plants and for developing future applications in stress diagnostics and precision crop management.

## Introduction

Research on plant–sound interactions is important not only in addressing a major unresolved question in plant sensory biology but also in connecting mechanobiology, ecology, and sustainable agriculture within a single conceptual framework. Plants live in vibrationally rich environments in which herbivore-associated vibrations, pollinator sounds, and anthropogenic noise can alter defense, reproduction, and physiological performance, while stressed plants themselves emit informative airborne ultrasonic signals that can be detected and classified. For this reason, phytoacoustics is significant at two complementary levels: fundamentally, to reveal how mechanical information is encoded, perceived, and transduced in plant systems; applied, to provide non-invasive tools for early stress diagnosis, precision irrigation, crop monitoring, and resilience-oriented management under drought and increasingly noisy agricultural conditions. In a cautiously forward-looking sense, this field also encourages us to reconsider plants not as silent background organisms, but as active participants in dynamic vibrational ecosystems—an idea that once belonged largely to speculative or philosophical discourse, but that is now becoming accessible to rigorous experimental testing through molecular, ecological, and bioacoustic methods.

### Sound as a physical and ecological component of plant environments

Sound is defined as a vibration that usually takes the form of an audible wave of pressure that propagates through a substrate (gas, liquid, solid). Each sound has its specific characteristics, such as frequency (Hertz, Hz), intensity or sound pressure level (watts per square meter, W/m^2^, or decibels, dB), propagation speed, and propagation direction.^1^ Although decibels (dB) are often used as a unit of perceived sound level and are popular in plant acoustic publications, watts per square meter (W/m^2^) seem more objective in describing studies investigating the effects of sounds on organisms. Therefore, we propose a unified way of reporting the sound intensity used in these studies by firstly describing it in W/m^2^, and only adding decibel values in brackets for easier comparison with other studies. Soundscape, understood as a sum of all the sounds occurring in a certain location in a specific timeframe, should be accounted as an inherent part of the abiotic environment of any considered ecosystem. As such, it plays an important role in communication, sensory cognition, and stress responses in almost all living organisms.

Plants, as sessile organisms inhabiting a range of different ecosystems, had to develop a variety of sensing mechanisms and response systems to abiotic and biotic stressors and stimuli. These include responses to light, temperature, water availability, osmotic changes, chemical compounds, and mechanical forces. Among mechanical stimuli, sound has historically received relatively little attention in plant science. However, both natural and anthropogenic environments expose plants to complex soundscapes composed of animal-generated sounds, abiotic background noise (such as wind, rain, and flowing water), and human-derived noise. These soundscapes vary in frequency, intensity, and temporal structure, creating dynamic mechanical environments that plants are continuously subjected to.^2^ Despite lacking specialized auditory organs analogous to those found in animals, accumulating evidence suggests that plants are capable of perceiving and responding to sound-related mechanical stimuli.^3^

Early interest in plant responses to sound dates back several decades and spans a spectrum from anecdotal observations to more systematic experimental approaches. Popular works such as *The Secret Life of Plants* and early experiments by Retallack suggested that plants might respond differently to various types of music, although these studies lacked rigorous experimental controls.^4^ ^,^ ^5^ On the other hand, the first reliable scientific reports of plants reacting to physical stimuli started to appear at the beginning of 20th century, long before the topic of plant–sound interactions was introduced to the mainstream literature. Foundational work by Bose demonstrated that plants are capable of generating electrical responses to external stimuli, establishing an early basis for the concept of plant sensitivity to physical signals.^6^ In the following years, controlled experimental studies began to explore the effects of sound waves on plant growth, reporting frequency-dependent changes in development.^7^ These early investigations, together with parallel efforts in agricultural contexts, laid the groundwork for later mechanistic studies, although reproducibility and methodological limitations remained significant challenges.

### Environmental sound interactions and plant responses

One of the most popular and compelling modern examples of plant responses to sound-related stimuli comes from plant–herbivore interactions. Plants exposed to playback of caterpillar chewing vibrations have been shown to activate herbivory-related defense mechanisms even before any physical damage occurs, indicating that vibrational cues alone are sufficient to trigger anticipatory responses.^8^ ^,^ ^9^

Similarly, plants are able to perceive pollinator-associated sounds. Exposure to buzzing frequencies characteristic of pollinators leads to an increase in nectar sugar concentration, enhancing attractiveness to insects. Notably, this response depends on the presence of floral structures, suggesting that plant morphology and geometry play an important role in resonance and sound perception.^10^ Mechanical vibrations are also central to buzz pollination, in which bees use body vibrations to extract pollen, and these vibrations are known to be amplified depending on flower structure.^11^ ^,^ ^12^

**Figure 1.**
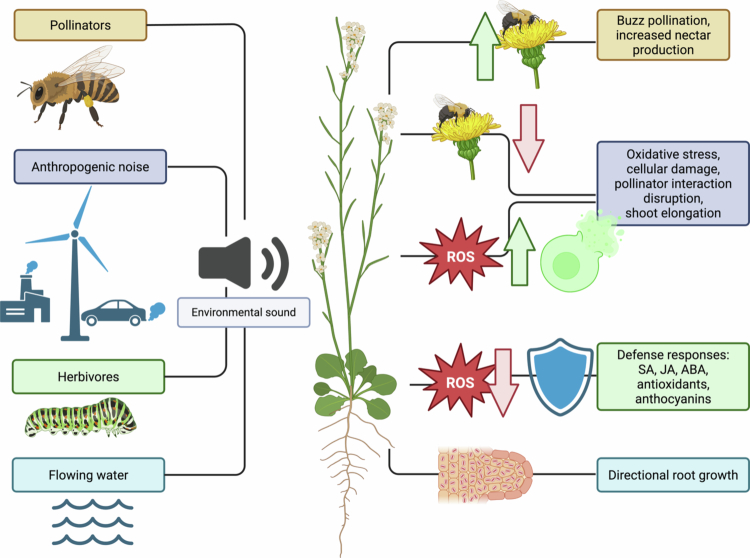
Sources of sound stimuli in plants' environments and their effects on plants' physiology.

Anthropogenic sound sources introduce an additional layer of complexity to plant soundscapes. Noise generated by human activity can influence both plants and their ecological interactions. For example, traffic noise has been shown to alter pollinator behavior, leading to reduced pollination efficiency in tomato plants.^13^ At the physiological level, exposure to anthropogenic noise can induce oxidative stress and potentially cause cellular damage.^14^ Low-frequency vibrations (<100 Hz, administered as substrate-borne stimuli through artificial shakers for 38 d) resembling those produced by wind turbines have been associated with increased shoot elongation in *Pisum sativum*, which may appear beneficial but could come at the cost of reduced defense responses and altered ecological interactions.^15^ The abundance of examples of plants being able to perceive and respond to environmental sounds highlights that this type of stimuli should be considered as an important part of plants' abiotic environment that significantly affects plants' functioning (Figure 1).

In addition to perceiving sound, plants may also produce sound as a response to mechanical damage or water stress.^16^ One of the most studied mechanisms underlying this phenomenon is cavitation in the xylem. When water columns within xylem vessels were subjected to negative hydrostatic pressure, the formation and collapse of air bubbles generate discrete acoustic emissions that are often described as clicking sounds.^17^ ^,^ ^18^ This has led to speculation that sound production could play a role in plant–environment or plant–plant interactions, although this remains poorly understood, and there is currently limited evidence that such emissions function as intentional communication signals rather than biophysical byproducts of hydraulic stress.

Taken together, the highlighted studies demonstrate that plants are not passive recipients of mechanical noise but can respond to complex acoustic environments and distinguish between different types of vibrational stimuli. Despite the growing body of evidence, the mechanisms underlying responses to sound remain incompletely understood. Key questions persist regarding the primary sites of sound perception, the identity of mechanosensitive components involved in signal detection and the pathways through which mechanical stimuli are translated into biochemical and transcriptional responses. It also remains unclear how plants discriminate between biologically relevant signals, such as herbivory vibrations and abiotic background noise, and whether acoustic signals can mediate communication between plants or between plants and other organisms.

The goal of this review is to investigate how plants perceive, process, and respond to sound stimuli. We synthesize current knowledge across multiple levels of biological organization, from physical signal propagation and mechanosensing to gene expression, metabolism, and whole-plant physiology. By integrating findings from ecological, physiological, and molecular studies, we aim to provide a comprehensive framework for understanding sound as a biologically relevant environmental factor in plant systems.

## Sensing mechanisms

The primary question that needs to be asked to enhance the understanding of plants' responses to sound is how they are able to perceive it in the first place. As shown in the studies presented in the Introduction, plants respond to both airborne and substrate-borne vibrations across a broad range of frequencies (<0.1–28 kHz). It was also shown that various plant organs react differently to specific types of sounds.^8-12^ We therefore hypothesize that plants may be able to sense sound as a mechanical vibration of the affected tissue, that is, as a series of repeated mechanical stimuli.

One of the best-studied candidates for a specialized mechanosensing structure are trichomes, which are small outgrowths of the epidermis present in the leaves and stems of many species. Mathematical modeling of Arabidopsis trichomes showed that their geometry enabled them to enter resonance in the presence of vibrations in the audible range, possibly matching with that of herbivore chewing sounds (best resonance response for 6–12 kHz).^19^ However, other parts of plants (such as flower petals and stigmas) were also shown to resonate and physiologically respond to sounds.^11^ ^,^ ^12^ Therefore, trichomes are unlikely to be the only sound-sensing structures in plants.

Baluška et al.^20^ provided one of the first attempts to describe a mechanistic model of sound sensing at the cellular level. They proposed that mechanical perturbations such as bending, vibration, or touch create strain in the cell wall and cytoskeleton. This strain is then detected by mechanosensitive proteins located at connections between the cell wall, plasma membrane, and cytoskeleton. The activation of these mechanosensors triggers intracellular signaling cascades, including calcium signaling, ion fluxes, and gene expression changes. Although the following studies tend to agree that mechanical stimuli are primarily transduced into plasma-membrane deformations, they generally focus more on the mechanosensitive ion channels present in the membrane rather than on the role of the cytoskeleton.^21^

Experiments with tobacco cell suspensions showed that sound exposure at different frequencies and intensities elevated cytosolic calcium levels and caused downstream cascades.^22^ Nakagawa et al.^23^ proposed the MCA1 protein (Mid1-complementing activity 1) as a likely candidate to play the main role in mechano-induced calcium influx. MCA1 was isolated by functional complementation of the lethal yeast mid1 mutant, a strain lacking a putative Ca^2+^-permeable stretch-activated channel component. The encoded protein is a 421-aa integral plasma-membrane protein that contains predicted transmembrane segments, an EF-hand-like motif, and a cysteine-rich PLAC8-related region. The expression of MCA1 partially rescued the mid1 lethality phenotype and increased Ca^2+^ uptake in yeast, even in the mid1 cch1 double knockout mutant, which is a phenotype which lacked both the main calcium channel (Cch1) and the supporting Mid1 complex, which would otherwise be unable to intake calcium from the environment. This suggests that MCA1 not only complements Mid1 but rather plays a distinct role in calcium homeostasis. In Arabidopsis, MCA1 was mainly localized to the plasma membrane of root cells, and it has been shown to improve calcium uptake in the roots. The effect of MCA1 was especially intense under hypoosmotic shock and trinitrophenol (TNP) treatment, both of which distorted the plasma membrane, indicating that MCA1-mediated calcium influx is triggered by plasma-membrane deformation.^23^ In the same publication, it was also demonstrated that MCA1 was crucial for the ability of roots to penetrate hard media, which suggests that MCA1 could also play roles in other processes related to mechanosensing in plants.^23^

Yamanaka et al.^24^ examined another protein, MCA2, which plays a role similar to that of MCA1. MCA2 was, conversely to MCA1, expressed in various locations across the plant and does not seem to play a role in the root's ability to penetrate hard media. However, its activity did overlap with that of MCA1 in terms of Ca^2+^ homeostasis. Under ionic stress, double (mca1-null, mca2-null) mutants were much more severely impaired than mca1-null mutants, which suggests that plant mechanosensitive Ca^2+^ uptake is not governed by a single channel but rather by a partially redundant system. Later, heterologous and reconstitution-based studies further supported this model: MCA1 expression enhanced mechanosensitive channel activity in *Xenopus laevis* oocytes, while purified MCA1/MCA2 fragments, when reconstituted into artificial liposomes, mediated Ca^2^⁺ influx and, in the case of MCA2, were directly activated by membrane tension.^25^ ^,^ ^26^ These findings indicate that MCA proteins do not require a fully plant-specific cellular environment to function as Ca^2^⁺-permeable mechanosensitive channels.

Although it might be tempting to view sound (and mechanical stimulation) perception as only Ca^2+^ -mediated, it seems that this system actually depends on multiple ion types. Maksaev and Haswell^27^ investigated MSL10 (Mechanosensitive ion channel like protein 10), which is activated by plasma-membrane tension and displays a clear preference for anions. Experiments with Arabidopsis-derived MSL10 in heterologous systems demonstrated that it closed at a much lower tension than that required for it to open, and under some conditions, can remain open even in the absence of further applied pressure. Another study linked MSL10 to cell-swelling-induced Ca^2+^ influx and a downstream increase in ROS production, as well as the expression of mechanosensing-related genes (TCH, WRKY) and cell-death-related genes (TUNEL, caspase-like activity).^28^ Since MSL10 has a clear preference for anions, this leads us to hypothesize that once the mechanical stimulus occurs, MSL10 allows for the transport of anions out of the cell to increase depolarization, which points towards a possible synergetic effect of MSL 10 and MCA1 and MCA2. Structural analysis^29^ confirmed that such activity would be energetically efficient since MSL10 tends to form pores that, without applied force, tend to remain open. It was also shown that channel closing occurs upon lipid displacement in the membrane, which causes a rotation of a single phenylalanine residue (F553) and the formation of a hydrophobic gate. This suggests an ultrasensitive and energy-efficient mechanosensing mechanism, where minimal structural movement produces large functional effects. Even though this mechanism still needs to be studied extensively, overall, it seems the response to mechanical stimuli is primarily based on changes in membrane polarization mediated by tension-activated ion channels.

Another interesting group of proteins likely to be involved in sound-related mechanosensing are PIEZO-like proteins. PZO1 (*Arabidopsis thaliana* PIEZO1) is strongly expressed in tissues expected to experience mechanical strain during root penetration, especially the columella and lateral root cap cells at the root tip.^30^ In columella cells, PZO1 contributes to mechanically induced Ca^2+^ transients, likely by functioning as, or forming part of, a mechanosensitive nonselective cation channel. This local Ca^2+^ signal helps roots adjust their penetration behavior, reduce maladaptive coiling, and continue growth through mechanically resistant substrates. Such studies highlight the need to further investigate how root function changes under sound-specific mechanostimulation and to identify what role PIEZO plays in those changes.

Apart from Ca^2+^ and anions' roles in mechanosensing, Zhao et al.^31^ demonstrated that the permeability of membrane K^+^ specific channels increased in *Oryza sativa* plants upon sound stimulation (0.01 W/m^2^ (100 dB), 1000 Hz, 30 min × 2 daily for 2 weeks). This confirms that mechanosensing mechanisms depend on a network of different ions.

In terms of primary sound sensing, it was also shown that the plasma membrane itself is affected upon treatment. Zhao et al.^32^ demonstrated that sound exposure (400 Hz optimal, 0.001–0.1 W/m^2^ (90–110 dB), 1 h) changed the secondary structure of protoplast membrane proteins in tobacco, leading to a decrease in β-turn content and an increase in the α-helix content. The structural alteration would, biophysically, cause membranes to become more flexible since higher β-turn to α-helix proportions are usually associated with greater membrane stability. Increased flexibility of the membranes could lead to a positive feedback loop, resulting in stronger activation of mechanosensitive (MS) channels upon further stimulation. Yi et al.^33^ also confirmed a similar set of changes (β-turn to α-helix proportion decreased) and directly showed that the fluidity of lipid barriers increased under sound treatment. The co-occurrence of changes in membrane properties and MS-channel activation from previous studies together confirm that the cell membrane is the main sensor for mechanical vibrations in plants.

Although many studies treat mechanical stimuli (touch) and sound waves similarly in terms of sensing mechanisms, the actual distinction (or lack thereof) remains unclear and is dependent on multiple factors. Ghosh et al.^34^ is the main study pointing to a difference between these two types of mechanostimulation. The expression of 13 MS ion channels was investigated in Arabidopsis after treatment either with sound (500 Hz, 0.01 W/m^2^ (100 dB) continuously for 5 d) or touch (bending leaves twice a day for 5 d). Results showed that the sound treatments upregulated MSL3, MSL4, MSL7, and MSL8 and downregulated MSL10, MCA2, and PIEZO; in contrast, the touch treatments upregulated MSL2, MSL3, MSL5, MSL6, MSL10, MCA1 and MCA2.^34^ This shows that, in long term mechanical treatments, the patterns of responses may differ between sound and touch. A continuous, 5-d-long stimulation is very intense compared to other studies. Therefore, we cannot rule out the possibility that a more similar expression pattern might occur after shorter periods of sound and touch stimulation.

## Downstream effects of primary ion dependent sound sensing

### Calcium sensing and related pathways

As suggested previously, calcium influx is the first step of the sound response in plants. Liu et al.^35^ broaden this view, showing that, upon sound exposure, calcium cations in the chrysanthemum callus tend to migrate from the vacuolar lumen to vacuolar membranes. The study also observed an increase in calcium levels in the cytosol, nucleus, chloroplasts and Golgi apparatus in treated cells, indicating that the sound-induced calcium changes were based not only on calcium influx from the extracellular environment but also to changes in the intracellular calcium distribution. This idea is further strengthened by the fact that sound stimulation increases the expression of calreticulin, which is a chaperone protein present in the ER that plays a key role in calcium storage and signaling, particularly during plant responses to stimuli.

Sound-induced growth effects resulted from sound causing a release of cell wall calcium and ion-channel dependent calcium influx into the cytoplasm and calmodulin-dependent signaling.^36^ Sound exposure (500 Hz) caused intensified transcription of both mechanosensitive genes (TCH4, DREB26) and calmodulin-like components (CML38), indicating that they might have an interconnected role in the sound response.^37^ As one of the possible primary effects of calmodulin signaling, Zhao et al.^38^ showed that sound stimulation significantly enhanced plasma membrane H⁺-ATPase activity in chrysanthemum callus, but only when intact signaling was present; the isolated membrane did not show the same autonomous response. These findings suggest that calmodulin downstream effectors and primary metabolic proteins may be important targets of subsequent sound‒response pathways in plants.

### ROS changes

Another primary sound response that seems to occur in parallel to calcium signalling-related changes is the ROS-related defense pathway. A range of anthropogenic noises cause negative effects on plants and tend to increase H_2_O_2_ levels in plant tissues, signifying oxidative stress.^3^ ^,^ ^14^ ^,^ ^15^ At the cellular level, upon sound treatment with a single frequency playback (500 Hz) ROS-related genes and defense genes (JAZ7, HSPRO2) were upregulated.^37^ Exposure of tobacco cells to a 1000 Hz single frequency treatment also increased reactive oxygen species such as H_2_O_2_ and caused membrane lipid peroxidation, indicating oxidative stress.^22^ The total antioxidant capacity and radical scavenging ability of the cells decreased under high-intensity sound treatment.^22^ Treatment of *Corylus avellana* (hazel) cells with low intensity ultrasounds was also shown to increase the levels of intracellular H_2_O_2._ ^39^ Upon treatment, an increased expression of catalase (CAT), the main antioxidative enzyme in plants, and phenylalanine ammonia-lyase (PAL), a key enzyme in the phenylpropanoid pathway associated with defense and secondary metabolite production, was observed. These results support a model in which ultrasound acts as a mechanical stimulus that initiates a controlled oxidative burst, which functions as a secondary messenger to activate stress-responsive metabolic and genetic pathways.

Across multiple studies of different plant species and after treatment with a range of sound frequencies and intensities, it was observed that expression, contents, and/or activity of several ROS scavenging and detoxifying enzymes were consistently increased including: superoxide dismutase (SOD),^37^ ^,^ ^40-45^ ascorbate peroxidase (APX),^37^ ^,^ ^40^ monodehydroascorbate reductase (MDAR),^37^ glutathione S-transferase (GST),^37^ dehydroascorbate reductase (DHAR), glutathione reductase (GR),^40^ and ferredoxin NADP+ reductase (FNR).^46^ Moreover, the levels of some secondary metabolites with antioxidant activity like crocin in saffron plants^47^ and general flavonoids in broccoli, alfalfa, radish, and lettuce sprouts were increased after specific sound treatments. Apart from the results presented in two studies,^45^ ^,^ ^47^ most of the experiments that resulted in increased ROS and/or higher antioxidant levels and activities were conducted using relatively low sound frequencies (up to 1000 Hz). This may indicate that sound treatment utilizing frequencies at the lower end of the spectrum can initiate a defense-like response in plants. This tendency, however, still requires more experimental data to be confirmed.

### Gene expression changes

The next logical downstream effect of sound stimulation and following calcium/ROS signaling are changes in gene expression in treated plants. Studies by Wang et al.^48^ performed on chrysanthemum seedlings treated with 1000 Hz 0.01 W/m^2^ (100 dB) artificial sound playback (1 h/d for 3–15 d) showed that upon treatment cells exhibited increased levels of RNA and soluble proteins but no changes in DNA content. The effect peaked at day 9 of treatment and decreased slightly on days 12–15. This suggests that sound has a significant, positive, time-dependent influence on the intensity of transcription and translation in plant cells but does not cause significant DNA damage (at least in a quantitative manner).

Work by Jeong et al.^49^ is one of the first studies linking the frequencies and durations of the sounds used for treatments to changes in gene expression. Their experiments demonstrated sound induced changes in the expression of multiple genes, including ald (aldolase) and rbcS (Rubisco small subunit). Aldolase was significantly upregulated upon treatment with 125 Hz and 250 Hz playbacks, while downregulated by 50 Hz. Duration was also important, ald expression peaked at 1 h post exposure and decreased thereafter. Other upregulated genes included calreticulin (mentioned above) and DNA-J-like protein, which is a chaperone protein responsible for abiotic stress management and chloroplast membrane stability.

Further studies utilizing newer -omics approaches^37^ demonstrated that sound at various frequencies (250–300 Hz) activated mechanosensitive genes (TCH4, DREB26), calcium signaling components (CML38), MAPK pathways, and large transcription factor networks (MYB, WRKY, ERF). Most of the upregulated genes were localized in the nucleus, and many were found to be transcription factors. The majority of the genes were classified as being involved in stress responses and signal transduction pathways. The proteomic analysis performed showed a strong increase in the expression of proteins involved in carbohydrate metabolism and photosynthesis, as well as defense mechanisms.^37^ Further, Kwon et al.^46^ found 38 proteins belonging to similar groups as those indicated by Ghosh et al.^37^ to be significantly altered after sound treatment. The changes were both time- and frequency dependent. The study showed that some stress related genes: Glutathione S-transferase, 2-Cys-Peroxiredoxin, Fe superoxide dismutase 1, manganese superoxide dismutase 1, and beta glucosidase 18, were upregulated at 8 h under 250 Hz and 500 Hz treatments, further suggesting that frequencies in the lower audible range may increase defense responses.

One of the newest transcriptomic data coming from experiments performed by Ye et al.^50^ using soft music playback (1 × 10^−6^−1 × 10^−5^ W/m^2^ (60–70 dB), 100–500 Hz, 5 h/d for 7 d) on *Lemna turionifera* (duckweed) indicated that, upon treatment, 537 genes were upregulated, and 759 downregulated. The highest number of upregulated genes were located in the areas of the chromosomes and the nucleoplasm. It was also observed that the highest number of downregulated genes were related to DNA metabolic processes (which corresponds logically to the fact that the DNA content tends not to vary upon sound treatment,^48^ ATPase activity, and GTPase activity. Most of the upregulated pathways were related to glycolysis, oxidative phosphorylation, the pentose phosphate pathway and cell wall biosynthesis. The type of music used in the experiment may cause allocation of energy and resources to metabolism and growth. This is generally contrary to most previous studies where single-frequency playbacks tended to cause an investment in defense mechanisms, suggesting the need for further research to look deeper into how differences in patterns and frequencies of sound treatment translate to differing results.

### Impact on metabolism

There are more reactions related to plant sensing that affect various metabolic processes. The simplest indicators of metabolic changes—increased ATP levels and higher activity of ATPases—were shown to occur in plants after various sound treatments. Sound stimulation significantly increased the ATP content in *Actinidia* callus.^51^ Interestingly, these changes seemed to be affected more by the frequency than the intensity of the treatment. Treating *Aloe arborescens* with ultrasound (20 kHz, for 2 s) caused an increase in vacuolar ATPase activity, H^+^ transport and ATP hydrolysis.^52^ There are many studies that have demonstrated specific sound treatments increased the expression of ATPase (or its specific subunits).^37^ ^,^ ^40^ ^,^ ^46^ Therefore, plants are able to translate primary sound sensing into changes in cellular energy allocation and economy.

Ghosh et al.^40^ showed high expression of genes related to cellular respiration, such as: triosephosphate isomerase, phosphoenolpyruvate carboxykinase, aldehyde dehydrogenase, pyruvate dehydrogenase, pyruvate kinase, and glyceraldehyde phosphate dehydrogenase, in plants that were infected by Botrytis and subjected to sound playback. This finding indicates that metabolic alterations caused by sound may be able to support plants already experiencing biotic stress, such as an infection.

Sound has also been shown to enhance anabolic processes in treated plants. Glutamate-glyoxylate aminotransferase 1 (GGAT), an enzyme involved in photorespiratory carbon and nitrogen metabolism, carbonic anhydrase (CA), which participates in CO₂/HCO₃^-^ interconversion and carbon assimilation, as well as several RuBisCO-related proteins, showed altered expression in response to sound treatments at different frequencies.^46^ Many of the differentially expressed proteins after sound exposure were associated with carbohydrate metabolism and photosynthesis.^46^

Some contrasting results are presented by Ghosh et al.^37^ They noted the upregulation of two large-chain members of RuBisCO (RBCL) in their analysis. However, three small subunits of RuBisCO (RBCS) were significantly downregulated by almost all the treatments they used, except 250 Hz. Uroporphyrinogen III decarboxylase (UROD), which is necessary for chlorophyll synthesis, was also upregulated after selected sound treatments. Phosphoribulokinase (PRK), an enzyme involved in the Calvin cycle, was upregulated 24–48 h after treatment with 3000 Hz. A light-harvesting complex protein (LHCB) was upregulated immediately after 250 Hz treatment, whereas the same protein was downregulated by other frequencies. These findings suggest that sound-induced changes in photosynthesis-related pathways are strongly dependent on treatment frequency and sampling time. They also indicate that different components of photosynthesis may not respond uniformly: RuBisCO large and small subunits, chlorophyll biosynthesis enzymes, Calvin-cycle enzymes, and light-harvesting proteins may each have distinct response windows.

In another study, two photosynthetic parameters were measured: Fv/Fm, which indicates the maximum quantum efficiency of photosystem II, and PI, which reflects the performance index for energy conservation from excitation to the reduction of PSI end acceptors.^34^ PI was significantly reduced after 5 d of sound treatment, although no significant change was observed in Fv/Fm. In contrast, Ye et al.^50^ demonstrated that Fv/Fm was significantly higher in sound-treated duckweed than in control-treated duckweed without music. The discrepancy between these results may reflect several experimental differences, including species identity (*Arabidopsis* versus duckweed), developmental and physiological state, sound regime (single-frequency treatment versus complex music playback), exposure duration, and light or growth conditions during treatment. This may also reflect the fact that photosynthetic gene/protein expression and chlorophyll fluorescence parameters do not necessarily change in parallel or on the same timescale.

Kwon et al.^46^ demonstrated the downregulation of PSI reaction-center subunit II2 and chlorophyll a/b-binding protein after 8 h treatments with 250 Hz and 500 Hz in *Arabidopsis*, while another study found that many genes were upregulated by sound treatment including photosystem I and II subunits, light-harvesting complex II chlorophyll a/b-binding proteins, ferredoxin-NADP⁺ reductase, and ribulose bisphosphate carboxylase.^40^ Importantly, the latter study was conducted in the context of *Botrytis cinerea* infection, meaning that sound treatment may have interacted with pathogen-induced metabolic reprogramming rather than acting on photosynthesis alone. Overall, these contrasting findings suggest that sound can influence photosynthesis-related pathways, but the direction and magnitude of the response likely depend on frequency, exposure duration, species, developmental stage, light environment, stress context, and whether the measured endpoint is transcript abundance, protein abundance, or physiological photosynthetic performance. More standardized experiments comparing multiple frequencies, species, growth conditions, and sampling times are therefore needed before firm conclusions can be drawn about the exact impact of sound on photosynthesis.

Secondary photosynthesis products and further biosynthetic processes also seem to be affected positively in plants treated with sound. Most of the starch synthesis related genes were upregulated in duckweed after exposure to soft music,^50^ while the soluble sugar content increased significantly under 1000 Hz 0.1 W/m^2^ (100 dB) 1 h/d after 14 d treatment of the callus of *Dendranthema morifolium.* ^53^ The biosynthesis of nitrogen-based compounds has also been shown to be enhanced after sound exposure. Aspartate-semialdehyde dehydrogenase (ASADH), the enzyme linked to the homoserine and threonine biosynthesis pathways, was up-regulated by 1000, 2000, and 3000 Hz in Arabidopsis.^37^ Similarly, cysteine synthase (CS) and S-adenosylmethionine synthase (SAMS), enzymes involved in the biosynthesis of cysteine and methionine, respectively, were strongly up-regulated by the three treatments. Moreover, Kwon et al.^46^ noted an upregulation of nitrogen metabolism-related genes after 8 and 24 h of 250 Hz and 500 Hz treatment, while nucleotide metabolism related genes (nucleoside diphosphate kinase and adenylate kinase), as well as seven genes related to amino acid metabolism, were upregulated in plants infected with Botrytis and treated with sounds.^40^ Finally, Zhao et al.^53^ showed that in *D. morifolium* callus subjected to 1000 Hz, 0.1 W/m^2^ (100 dB), 1h/day 14 d treatment, there was an overall increase in the level of soluble proteins.

The abundance of the known positive effects of sound treatment on biosynthesis should be perceived as relevant not only in terms of the altered biochemistry of the treated plants but also in a broader perspective. Based on all the positive results discussed in the paragraphs above we hypothesize that in the presence of curated sound treatment, edible crops might be able to achieve increased nutritional values. Also, from an ecological point of view it would be worth considering in future studies whether specific sounds might be perceived by wild plants as a signal to increase their nutrient production in a beneficial environment during a specific period.

## Phytohormones

### Auxins

As shown earlier, sound treatment can alter both signaling pathways and biosynthetic processes in plant cells. In that context, we believe it is reasonable to examine sound-induced changes in phytohormone production and homeostasis.^37^ ^,^ ^54^ Firstly, there is clear evidence that sound treatment at specific frequencies can affect the auxin content in certain species. Treatment with 1400 Hz, 0.00316 W/m^2^ (95 dB), 1 h/d increased IAA levels in chrysanthemum callus, with the strongest effects observed on treatment days 10–12.^54^ Furthermore, in *Actinidia chinensis* callus treated with mechanical vibration at low frequencies, the strongest reported effect were around 3 Hz, which reduced IAA oxidase activity. Since IAA oxidase is involved in auxin degradation, this reduction may have contributed to higher endogenous IAA availability, although IAA levels were not directly measured, and may partly explain the observed stimulation of callus growth^43^. Very similar effects were noted by Liu et al.^41^ after treating chrysanthemum callus with sound at a lower frequency of 800 Hz, 0.1 W/m^2^ (100 dB), 60 min/d for 10 d. This suggests that, to achieve an increase in IAA levels in different plant species, it is necessary to calibrate the frequency and duration of exposure to the plant species.

Additionally, the effects of ultrasound treatment were reported by Wei et al.^45^ IAA level decreased in protocorm-like bodies of *Dendrobium officinale* treated with ultrasound (28 kHz, 300 W, 5 min), whereas the levels of isopentenyladenine and isopentenyladenine-9-riboside, both of which are cytokinins, increased. The levels of zeatin and zeatin riboside were also slightly higher under ultrasound treatment. The overall CTK-to-IAA ratio increased after treatment. Under the same conditions, IAA oxidase levels increased, and CTK oxidase levels decreased.^45^ This indicates that, unlike some selected lower-frequency treatments, ultrasound may reduce auxin levels and shift the hormonal balance towards cytokinins.

### Jasmonic acid

Another class of phytohormonal changes following sound treatment is associated with defense mechanisms. Body et al.^9^ examined phytohormonal changes after treating plants with either playbacks of herbivore chewing vibrations, methyl jasmonate (MeJa; a volatile compound used by plants to signal herbivory danger), or a combination of both. Vibration playback alone caused an overall decrease in the content of the studied hormones, but the effects differed when MeJa was introduced. When compared with plants treated with MeJa alone, the combination of MeJa-treatment with vibration treatment enhanced defense responses by significantly increasing the levels of jasmonic acid (JA) and indole-3-butyric acid (IBA, an auxin involved in defense mechanisms). This may suggest that herbivory-resembling vibrations do not necessarily elicit a defense response on their own but may enhance and complement the effects of other primary herbivory defense elicitors, such as MeJa. On the other hand, Ghosh et al.^37^ observed that single-frequency playback at 500 Hz, without any additional stress elicitors, caused an increase in salicylic acid (SA), gibberellins, and auxins at 24 h post-treatment, and an increase in JA at 48 h. This indicates, firstly, that specific sound frequencies can elicit hormonal stress responses on their own and, secondly, that the profiles of these responses are strongly time dependent.

### Salicylic acid

Kwon et al.^46^ and Choi et al.^55^ also used single-frequency treatment to initiate a hormonal stress response, although in a different context. They showed that, after 1000 Hz treatment and *B. cinerea* infection, plants produced more SA than infected plants not exposed to sound, whereas JA remained unchanged or was slightly reduced. This suggests that sound treatments at certain frequencies can support plants experiencing different types of biotic stress by enhancing the hormonal response profile needed to mitigate a specific stress.

### Abscisic acid

Interestingly, another hormone involved in plant stress management, abscisic acid (ABA), seems to display a different pattern in response to sound treatment. ABA levels remained unchanged across the different time points (0–48 h) and the five tested frequencies.^37^ Similarly, the ABA levels in chrysanthemum callus treated with 1400 Hz, 0.00316 W/m^2^ (95 dB), 1 h/d did not differ significantly from those in the control and then decreased significantly at day 10.^54^ These findings may suggest that sound does not induce a generic stress state in plants but rather elicits responses resembling those associated with specific environmental challenges, like herbivory, or infections.^9^ ^,^ ^37^ ^,^ ^46^ ^,^ ^55^

### Ethylene

Ethylene is another important phytohormone, in which sound-induced changes are reported mainly in fruits and seeds rather than in other organs. In tomato fruits treated with 1 kHz at 0.01 W/m^2^ (100 dB) for 6 h postharvest, ethylene levels were significantly lower than those in the control.^56^ LeACS2, LeACS4, and LeACO1 (ethylene biosynthesis-related genes) were also downregulated in treated fruits. E4 and E8 (ethylene-inducible genes) were likewise significantly downregulated by sound treatment. Moreover, LeHB-1 and TAGL1, upstream transcription factors related to ethylene biosynthesis, were downregulated. NOR, a transcription factor related to fruit ripening and to ethylene and carotenoid biosynthesis, was also significantly downregulated after treatment. The expression levels of RIN, a master transcription factor regulating ripening, as well as ACS2 and ACS4, also remained low under treatment.^56^ Another study showed that ethylene is crucial for Arabidopsis seeds to respond positively to vibration stimulation, as mutants deficient in ethylene production showed no increase in germination under sound treatment.^57^

Overall, these findings indicate that sound-induced phytohormonal responses depend strongly on multiple factors, including species, intensity, frequency, exposure pattern, and treatment context. With further extensive studies, the complexity of these hormonal responses could potentially be utilized in the future to design targeted, species-specific treatment regimens that help plants mitigate stress or enhance desirable physiological traits.^54^ ^,^ ^56^ ^,^ ^58^

## Plant phenotypes

### Growth enhancement

Knowing that sound treatment can positively affect both biosynthetic processes and phytohormone levels, we turned our attention to whether sound can also improve the growth patterns and growth efficiency of plants.^53^ ^,^ ^54^ ^,^ ^59^ Starting with tissue culture experiments, treatment with 1400 Hz, 0.00316 W/m^2^ (95 dB), and 1 h/d increased biomass accumulation in chrysanthemum callus after approximately 10 d.^54^ Similarly, the growth rate was also increased after treatment with 1000 Hz, 0.01 W/m^2^ (100 dB), 1 h/d for 14 d in callus of *D. morifolium.* ^53^ Fresh weight increase was also reported in paddy rice plants exposed to a range of sound treatments, with 400 Hz at 0.04 W/m^2^ (106 dB) producing the best results.^59^ Ultrasound exposure produced an increase in the fresh weight of *Aloe arborescens* suspension-cultured cells when the treatment lasted 2 or 5 s and a decrease when the exposure was longer (10–60 s).^52^ Interestingly, in callus of *A. chinensis*, which is a woody plant, a significant increase in fresh weight was achieved at a higher frequency (3 kHz; tested range 1–5 kHz) than in other plants examined in the same laboratory.^43^ In non-woody plants, a similar positive effect was achieved at much lower frequencies.^53^ ^,^ ^54^ ^,^ ^59^suggesting that species with mechanically firmer tissues require higher treatment frequencies to achieve positive biomass responses. Some studies indicate that certain sounds can disrupt growth, for example, traffic noise can negatively affect the growth of urban plants by inducing oxidative stress.^14^ Negative effects on biomass gain were also observed in solid callus cultures of *Aloe arborescens* exposed to the highest ultrasound intensities (5–10 W).^52^

There is also evidence of a significant effect of sound treatment on shoot elongation.^45^ ^,^ ^59^ Plant height in paddy rice increased significantly (~15%–20%) after a range of sound treatments, with 400 Hz at 0.04 W/m^2^ (106 dB) being the most beneficial.^59^ Shoot elongation increased in rice coleoptiles but not in cucumber hypocotyls when both were treated with 50 Hz vibration for 3 d.^60^ This suggests that shoot elongation responses to specific frequencies are species dependent. Protocorm-like bodies of *Dendrobium officinale* showed increased differentiation into shoots when treated with 28 kHz ultrasound, indicating that ultrasound may not only stimulate shoot growth but also alter resource allocation towards shoot tissue development.^45^ This pattern may be related to the phytohormonal shifts discussed earlier in this review.^41^ ^,^ ^43^ ^,^ ^54^ Roots also seem to be affected by sound vibrations. The root number, total root length, and root activity (TTC assay for cellular metabolic activity) increased in paddy rice exposed to a range of sound treatments.^59^ Root elongation also increased in cucumber and rice seedlings treated with 50 Hz vibration for 3 d.^60^

One explanation for the overall positive effects of sound on growth rates and biomass accumulation in different plant tissues may lie in sound-induced alterations of the cell wall. Sound altered the activities of cell-wall-modifying enzymes in tobacco cells, suggesting structural remodeling of the cell wall in response to vibration.^22^ Similarly, Ghosh et al.^40^ identified at least four genes related to cell walls that responded to sound: TCH4, laccase12, GOLS8, and XTH18. TCH4 encodes a cell-wall-modifying enzyme that breaks xyloglucan chains and makes the cell wall more elastic, which could facilitate cell elongation. Pectin synthesis and lignin deposition were also positively affected by sound treatments at various frequencies,^22^ ^,^ ^50^ which may be among the causes of sound-induced biomass accumulation.

### Stress response, priming, and other physiological applications

Apart from the influence of sound on yield, there is also evidence for other beneficial effects of this type of treatment, including improved stress tolerance. Sound treatment can mitigate the negative effects of water scarcity on plants. Drought-experiencing rice plants subjected to different sound treatments (0.25–1.5 kHz, 0.01 W/m^2^ (100 dB), 1 h) showed improved adaptation to water deficiency, including decreased osmotic potential, increased dark-adapted quantum yield, decreased H_2_O_2_ levels (linearly and inversely proportional to the tested frequencies), and increased stomatal conductance.^58^ This shows that sound vibration treatment can shift plant physiology towards greater stress resistance. Similarly, Arabidopsis after sound treatment had significantly higher survival rates under drought.^61^ A possible explanation may lie in improved ROS management, which is crucial for drought responses. The previously mentioned increased JA levels in response to sound treatment might also contribute, because there is evidence that JA takes part in the overall plant drought stress response.^62^ However, this assumption needs to be tested experimentally, as there are no studies directly measuring JA in plants experiencing drought and treated with sound.

These findings allow us to hypothesize that sound treatment may have agricultural potential for reducing yield losses in drought-affected regions. This is particularly relevant because increasingly unpredictable climate patterns are intensifying the need for environmentally friendly methods that support crops and help secure food production.

Another area in which sound treatment may be useful is the mitigation of plant infections. Sound-treated Arabidopsis infected with Botrytis showed stronger defense responses, including higher antioxidant capacity, higher metabolic throughput, and reduced senescence.^40^ Among the many proteomic changes observed upon treatment, some of which we have already mentioned, there were clear increases in defense-related proteins such as lectins, VSP1, cysteine protease inhibitors, and ESM1. These changes correspond well with the observation that some sound treatments positively affect SA levels, a phytohormone crucial for pathogen responses. Although the plant-protective properties of sound treatment remain understudied, we anticipate that with increased research on this topic, this type of treatment may find applications in the field of ecological agriculture in the future.

### Impact on reproductive processes

As previously described in the introductory text, reproductive tissues are sensitive to environmental sound, especially anthropogenic noise and the buzz of pollinators. Buzz vibrations can enhance nectar sugar content and facilitate buzz pollination, whereas traffic noise can disrupt pollination and flowering processes.^10-12^ For flowering *per se*, Razavizadeh and Ziaratnia^47^ provided evidence that ultrasound treatment of saffron corms can significantly improve flowering efficiency.

Artificial single-frequency playback has also been used to control ripening rates. Ripening in sound-treated tomato fruits was delayed relative to the untreated control.^56^ Under continuous 1 kHz treatment, the effect was strongest: respiration-rate patterns were consistent with delayed ripening, fruit firmness was higher, and fruits remained green for longer. As discussed, when analyzing the same study in the phytohormones section, this phenomenon was strongly linked to sound-induced changes in ethylene biosynthesis.

Further, sound treatment was also shown to improve the germination processes. Germination rates of Arabidopsis seeds increased with the rising acceleration calculated from the vibration waves' amplitude and frequency.^57^ This response is most probably dependent on amyloplasts (since there was no response in mutants lacking starch) and on ethylene (no response in ethylene insensitive mutants). Similarly, Takahashi et al.^60^ demonstrated that the germination rates of sprouting rice and cucumber seeds were increased at approximately 30 h of vibration exposure (50 Hz).

### Nutrient content changes

As discussed in the metabolism section, sound treatment can have a significant effect on biosynthetic processes. This indicates the potential for sound to improve not only growth but also nutrient accumulation and, therefore, the agricultural value of crops. Measures and Weinberger^63^ examined the effects of sound treatment on one of the most agriculturally important species worldwide—*Triticum sativum*. In the grains treated with 5 kHz, 0.00158 W/m^2^ (92 dB) for 4 weeks, there was no change in total amino acid content, although the proportions of specific amino acids changed: alanine was higher in the treated embryos and lower in endosperm, glycine and alanine were consistently higher overall, and the asparagine content was lower in the sonicated endosperm tissue. This suggests that specifically designed sound treatment may have the potential to modify amino acid profiles in specific plant species. In other species, however, sound may impact not only amino acid composition but also total protein content. For example, the protein content in duckweed treated with music for 7 d was significantly higher than that in control plants.^50^

Some studies also reported increased micronutrient contents in sound-treated plants. The ascorbic acid content of alfalfa sprouts can be increased by applying specific sound frequencies at appropriate times.^44^ Overall, flavonoid contents increased in some vegetable sprouts (alfalfa, broccoli, radish) and decreased in others (lettuce, Chinese cabbage) under different sound frequencies and treatment durations.^64^ ^,^ ^65^ The characteristics of effective treatments varied substantially across species, suggesting that sound regimes would need to be tailored to the treated crop. We also hypothesize that, because photosynthesis was intensified in many of the species discussed in the metabolism section, the sugar content and overall energy value could be increased in sound-treated edible crops.^20^ ^,^ ^37^ ^,^ ^50^ ^,^ ^53^ However, the subject of sound-induced nutritional recomposition still requires extensive research.

## Summary, limitations, and perspective

**Figure 2.**
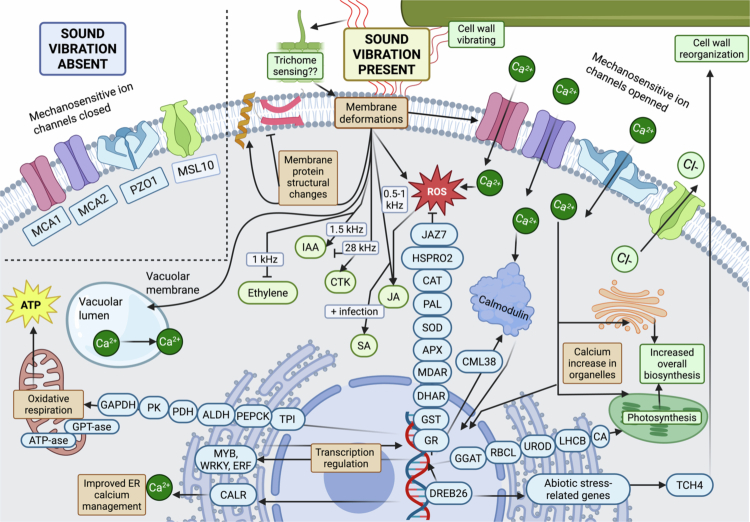
Sound-induced mechanosensing mechanism and its downstream effects. Sound vibration (possibly detected through cell wall vibrations transduction or through trichomes) causes membrane deformations which result in mechanosensitive ion channels opening. Resulting calcium cations influx causes calcium storage alterations in the cell, an ROS-related response, and the calmodulin pathway. This triggers the cell to alter the expression of genes involved in oxidative respiration, photosynthesis, defense response, and antioxidant activity. Sound also alters the levels of selected phytohormones, depending on the frequency.

Taken together, the literature reviewed here supports the conclusion that sound and vibration can act as biologically meaningful mechanical inputs that reshape plant signaling, hormone balance, and whole-plant performance (Figure 2 and Table 1). The strongest pattern emerging from the evidence is not that plants respond to “sound” in one uniform manner, but that they respond to a broad and highly heterogeneous class of mechanical wave stimuli whose biological consequences depend on stimulus physics, delivery route, species identity, developmental stage, and physiological context. This distinction is essential. Across the studies summarized in this manuscript, beneficial outcomes were repeatedly reported for biomass accumulation, shoot and root growth, germination, drought resilience, pathogen defense, metabolite accumulation, and post-harvest ripening control; however, these outcomes were rarely produced by a single conserved treatment window. Instead, the field points to stimulus-specific and species-specific response landscapes, suggesting that acoustic treatments behave more like precise elicitors than universal growth promoters.

**Table 1.** Topic-based overview of plant responses to sound and vibration.

Plant response topic	Effects observed	Sound treatment parameters reported in the review	Plant species/material
Primary sensing and ion flux	Ca2+ elevation and intracellular Ca2+ redistribution;^22^ ^,^ ^35-37^ K+ channel permeability increased^31^; plasma-membrane proteins/lipids became more flexible^32^ ^,^ ^33^; sound and touch produced different mechanosensitive-channel expression profiles^34^	Different frequencies/intensities^22^; 1000 Hz, 0.01 W/m^2^, 30 min twice daily for 2 weeks^31^; 400 Hz optimal, 0.001–0.1 W/m^2^, 1 h^32^; 500 Hz, 0.01 W/m^2^, continuous 5 d^34^; 500 Hz in signaling assays^37^	Tobacco cells^22^ ^,^ ^32^; chrysanthemum callus/roots^33^ ^,^ ^35^ ^,^ ^36^; Oryza sativa^31^; Arabidopsis^34^ ^,^ ^37^
ROS and antioxidant response	ROS/H_2_O_2_ and lipid peroxidation increased under some regimes^22^ ^,^ ^39^; high-intensity sound reduced antioxidant capacity^22^; CAT, PAL, SOD, APX, MDAR, GST, DHAR, GR and FNR activity/expression increased^37,39–44,46^; crocin/flavonoids increased in selected species^47^ ^,^ ^64^ ^,^ ^65^	500 Hz^49^; 1000 Hz^22^; low-intensity ultrasound^39^; 250–300 Hz^37^; 250–500 Hz, 8–24 h^46^; ultrasound in saffron^47^; species-specific audible regimes for sprouts^44^ ^,^ ^64^ ^,^ ^65^	Tobacco cells^22^; hazel cells^39^; Arabidopsis^37^ ^,^ ^40^ ^,^ ^46^ ^,^ ^49^; chrysanthemum^41^ ^,^ ^42^; alfalfa, broccoli, radish, lettuce and other sprouts^44^ ^,^ ^64^ ^,^ ^65^; saffron^47^
Gene expression and proteome	RNA and soluble protein increased without DNA-content change^48^; ald, rbcS, calreticulin and DNA-J-like protein changed^49^; TCH4, DREB26, CML38, MAPK and MYB/WRKY/ERF networks activated^37^; stress, photosynthesis and carbohydrate-metabolism proteins altered^37^ ^,^ ^46^; music changed 1296 duckweed genes and upregulated energy/cell-wall pathways^50^	1000 Hz, 0.01 W/m^2^, 1 h/d for 3–15 d^48^; 50/125/250 Hz^49^; 250–3000 Hz^37^; 250/500 Hz, 8–24 h^46^; soft music 100–500 Hz, 1e-6-1e-5 W/m^2^ (60–70 dB), 5 h/d, 7 d^50^	Chrysanthemum^48^; Arabidopsis^37^ ^,^ ^46^ ^,^ ^49^; Lemna turionifera/duckweed^50^
Phytohormones	IAA increased or IAA oxidase decreased under selected audible/low-frequency treatments^41^ ^,^ ^43^ ^,^ ^54^; ultrasound decreased IAA and shifted balance toward cytokinins^45^; herbivore vibrations altered hormones/volatiles and enhanced MeJA-induced JA/IBA^9^; 500 Hz increased SA, gibberellins, auxins and later JA^37^; 1000 Hz + Botrytis increased SA^46^ ^,^ ^55^; ABA was unchanged or decreased^37^ ^,^ ^54^; ethylene biosynthesis/signaling was downregulated, delaying tomato ripening^56^	1400 Hz, 0.00316 W/m^2^, 1 h/d^54^; 800 Hz, 0.1 W/m^2^, 60 min/d, 10 d^41^; low-frequency vibration, strongest around 3 Hz^43^; 28 kHz ultrasound, 300 W, 5 min^45^; chewing-vibration playback ± MeJA^9^; 500 Hz^37^; 1000 Hz + *B. cinerea* ^46^ ^,^ ^55^; 1 kHz, 0.01 W/m^2^, 6 h postharvest^56^	Chrysanthemum and Actinidia callus^41^ ^,^ ^43^ ^,^ ^54^; Dendrobium protocorm-like bodies^45^; Arabidopsis^9^ ^,^ ^37^ ^,^ ^46^ ^,^ ^55^; tomato fruit^56^
Metabolism and photosynthesis	ATP content and ATPase activity increased^51^ ^,^ ^52^; carbohydrate metabolism, respiration, carbon fixation and photosynthesis-related genes/proteins changed^37^ ^,^ ^40^ ^,^ ^46^ ^,^ ^49^ ^,^ ^50^; Fv/Fm or performance index responses differed by treatment/species^34^ ^,^ ^50^; and starch synthesis, soluble sugars/proteins, amino-acid and nitrogen metabolism increased or shifted^46^ ^,^ ^49^ ^,^ ^50^ ^,^ ^53^	Varied sound stimulation; frequency dominated ATP response in Actinidia^51^; 20 kHz ultrasound, 2 s^52^; 250–3000 Hz^37^; 250–500 Hz^46^; music 100–500 Hz, 5 h/d, 7 d^50^; 1000 Hz, 0.1 W/m^2^, 1 h/d, 14 d^53^; 1000/2000/3000 Hz^49^	Actinidia and Aloe callus/cells^51^ ^,^ ^52^; Arabidopsis^37^ ^,^ ^40^ ^,^ ^46^ ^,^ ^49^; duckweed^50^; *Dendranthema morifolium* callus^53^
Growth, biomass and architecture	Biomass/fresh weight and growth rate increased^43^ ^,^ ^53^ ^,^ ^54^ ^,^ ^59^; plant height, shoot elongation, root number, root length and root activity increased in responsive species^59^ ^,^ ^60^; Dendrobium shoot differentiation increased^45^; and excessive ultrasound or traffic noise inhibited growth^14^ ^,^ ^52^	1400 Hz, 0.00316 W/m^2^, 1 h/d, about 10 d^54^; 1000 Hz, 0.01–0.1 W/m^2^, 1 h/d, 14 d^53^; 400 Hz, 0.04 W/m^2^ best in rice^59^; 3 kHz best in Actinidia callus^43^; 50 Hz vibration for 3 d^60^; 28 kHz ultrasound, 300 W, 5 min^45^; 20 kHz ultrasound 2–5 s beneficial but 10–60 s or 5–10 W harmful^52^	Chrysanthemum/Dendranthema and Actinidia callus^43^ ^,^ ^53^ ^,^ ^54^; paddy rice^59^; rice and cucumber seedlings^60^; Dendrobium^45^; Aloe cells/callus^52^; urban plants^14^
Stress tolerance, priming and defense	Drought adaptation improved: lower osmotic potential, higher dark-adapted quantum yield, lower H2O2, higher stomatal conductance and/or higher survival^58^ ^,^ ^61^; Botrytis response improved via antioxidant capacity, metabolic throughput, reduced senescence and defense proteins^40^ ^,^ ^55^; herbivore-chewing vibrations primed defense signaling^8^ ^,^ ^9^; and anthropogenic noise could induce oxidative stress or alter development/herbivory^14^ ^,^ ^15^	0.25–1.5 kHz, 0.01 W/m^2^, 1 h in droughted rice^58^; sound treatment in Arabidopsis drought assays^61^; sound + B. cinerea, including 1000 Hz context^40^ ^,^ ^55^; herbivore-chewing vibration playback^8^ ^,^ ^9^; traffic noise^14^; <100 Hz wind-turbine-like vibration^15^	Rice^58^; Arabidopsis^8^ ^,^ ^9^ ^,^ ^40^ ^,^ ^55^ ^,^ ^61^; two urban plant species^14^; plants exposed to anthropogenic vibration/herbivory context^15^
Reproduction, ripening and germination	Pollinator buzz increased nectar sugar within minutes and flower structures amplified buzz-pollination vibrations^10–12^; traffic noise reduced tomato reproductive success^13^; ultrasound improved saffron flowering efficiency^47^; sound delayed tomato ripening and maintained firmness/green color^56^; and germination increased in Arabidopsis, rice and cucumber^57^ ^,^ ^60^	Pollinator/buzzing frequencies^10-12^; traffic noise^13^; ultrasound treatment of saffron corms^47^; 1 kHz, 0.01 W/m^2^, 6 h postharvest^56^; vibration acceleration treatments in Arabidopsis seeds^57^; 50 Hz vibration, about 30 h for germination or 3 d seedling exposure^60^	Flowers/pollinator-interacting plants^10-12^; tomato^13^ ^,^ ^56^; saffron corms^47^; Arabidopsis seeds^57^; rice and cucumber seeds/seedlings^60^
Nutrients and edible metabolites	Wheat amino-acid profile shifted without changing total amino acids^63^; duckweed protein increased^50^; alfalfa ascorbic acid increased^44^; total flavonoids increased in alfalfa, broccoli and radish but decreased in lettuce/Chinese cabbage under some regimes^64^ ^,^ ^65^; and saffron crocin/stigma metabolites were altered^47^	5 kHz, 0.00158 W/m^2^ (92 dB), 4 weeks^63^; music 100–500 Hz, 1e-6-1e-5 W/m^2^, 5 h/d, 7 d^50^; species-specific audible sound frequencies/timing for ascorbate/flavonoids^44^ ^,^ ^64^ ^,^ ^65^; ultrasound corm treatment^47^	*Triticum sativum*/Rideau wheat^63^; *Lemna turionifera* ^50^; *Medicago sativa*, *Brassica oleracea*, *Raphanus sativus*, lettuce and Chinese cabbage sprouts^44^ ^,^ ^64^ ^,^ ^65^; *Crocus sativus* ^47^

Note: Citation numbers refer to the manuscript reference list. Where exact frequency, intensity, or duration was not specified in the review text, the table preserves the review's qualitative treatment descriptor only.

A major conceptual advance is to place plant acoustic responses within the wider framework of plant mechanobiology rather than treating them as isolated phenomenon. Recent reviews of mechanostimulation have shown that vibration and sound belong to the same continuum as touch, wind, rain, bending, and other mechanical cues that trigger calcium influx, reactive oxygen species dynamics, phytohormone reprogramming, transcriptional shifts, cell-wall remodeling, and eventually altered morphogenesis.^66^ In that context, many of the effects described in the present review, including changes in auxins, jasmonates, salicylates, ethylene-related ripening genes, root activity, biomass accumulation, and defense competence, are biologically plausible. They are consistent with the view that acoustic treatments are converted into biochemical information through mechanoresponsive membranes, cell walls, ion channels, and downstream signaling networks. At the same time, mechanobiology also warns against overinterpretation: a response to vibration does not by itself imply specialized acoustic communication, and plant responses to airborne sound, substrate-borne vibration, and ultrasound should not be treated as interchangeable.

This point is especially important for interpreting phytohormone data. The hormonal outcomes described in the auxin, defense, ABA, and ethylene sections do not support a simplistic model in which sound acts as a generic stressor. Instead, the hormonal signatures appear to be selective and context dependent. In some systems, specific audible frequencies increased IAA content or reduced its degradation, which is consistent with enhanced cell expansion and biomass accumulation. In other systems, especially after ultrasound treatment, auxin levels declined while cytokinin-related compounds increased, indicating that different acoustic regimes may redirect developmental allocation rather than merely amplify growth. The defense-associated responses are equally nuanced: herbivory-like vibrations alone were not always sufficient to induce a strong defense profile, but they could potentiate methyl jasmonate responses, while single-frequency treatments in other pathosystems altered SA or JA accumulation in ways that better matched the encountered stress. This pattern suggests that acoustic stimulation may function as a modulatory signal that sensitizes existing defense networks, changes hormonal thresholds, or biases crosstalk among the JA, SA, auxin, and ethylene pathways rather than acting upstream of all defense responses in the same manner.

The physiological and yield-related observations further reinforce a hormetic interpretation of sound treatment. Low or moderate exposures often improved callus growth, seed germination, shoot elongation, root architecture, drought tolerance, chlorophyll retention, antioxidant capacity, and, in some cases, tissue differentiation or delayed fruit ripening. Yet stronger or longer treatments, traffic noise, or certain ultrasound intensities could impair growth or induce oxidative damage. Such bidirectional behavior is characteristic of mechanical dose dependence: the same class of cue can be stimulatory within one window and disruptive outside it. For agricultural translation, this is both promising and cautionary. It means that sound cannot be deployed as a fixed recipe; instead, the frequency content, sound pressure or vibrational amplitude, exposure duration, repetition schedule, coupling medium, and developmental timing must be optimized for each crop and objective. From a translational perspective, the most realistic near-term uses are those in which the target trait is narrow and measurable: seed priming, micropropagation and tissue culture, nursery-stage stress conditioning, post-harvest ripening management, and the enhancement of selected phytochemicals or defense readiness. Earlier application-focused syntheses also emphasized that sound-wave effects depend strongly on frequency, sound pressure level, exposure period, distance from the source, and cultivation system, with reported yield effects varying across greenhouse and field crops.^67^

Our work also highlights an emerging but still underdeveloped idea: sound-based treatments may be valuable not only because they modify the plant itself but also because they may influence the plant holobiont. Recent perspectives have argued that acoustic stimuli should be studied at the level of the plant–soil–microbe system, particularly where root physiology, rhizosphere signaling, and microbial dynamics intersect. This is an important frontier because many agronomically desirable outcomes—improved nutrient acquisition, drought buffering, induced resistance, and metabolic reprogramming—are not purely plant-autonomous traits. If sound alters root exudation patterns, membrane transport, root growth geometry, or microbial activity, then part of the observed benefit may emerge from a reconfigured rhizosphere rather than a direct plant-only effect. At present, however, this remains more of a hypothesis than an established mechanism, and future studies should explicitly include microbiome-resolved designs rather than treating soil as an inert background.

At the same time, several major gaps prevent the field from moving from intriguing case studies to predictive biology. The first unresolved question concerns perception. There is now abundant mechanistic evidence from broader plant mechanosensing research that mechanical cues can trigger local and systemic calcium and electrical signaling through mechanosensitive channels and related membrane systems, and recent work has strengthened the role of MSL-type channels and other mechanosensors in plant touch perception. However, no consensus receptor system has yet been demonstrated for sound vibration as such. It therefore remains unclear whether plants detect acoustic stimuli through dedicated frequency-sensitive structures, through general membrane tension and cell-wall deformation, through resonance-like properties of particular tissues, or through combinations of these processes. The second unresolved question concerns stimulus identity. Many studies still conflate audible airborne sound, substrate-borne vibration, ultrasound, music, and environmental noise, even though these stimuli differ profoundly in propagation physics, tissue coupling, attenuation, and likely biological entry points. Without cleaner stimulus taxonomy, the field will struggle to compare datasets or identify generalizable principles.

A third limitation is methodological standardization. Future experiments should report, at minimum, the spectral profile of the applied stimulus, calibration at the level of the plant organ rather than only at the speaker, background noise, chamber acoustics, distance and orientation to the emitter, coupling medium, exposure periodicity, and real vibrational amplitude experienced by the tissue. Sham controls also need improvement. For example, any apparatus that produces heat, airflow, electromagnetic artifacts, or incidental substrate movement can confound interpretation. A fourth challenge is the biological timescale. Many studies focus on early transcriptional or short-term phenotypic responses, but far fewer address persistence, stress memory, developmental trade-offs, reproductive consequences, or multi-generational effects. This is crucial if sound is to be developed as a priming technology. Mechanical priming in crops is increasingly discussed in the wider mechanobiology literature, yet whether sound-induced acclimation is mediated by transient physiology, durable structural remodeling, transcriptional memory, or epigenetic marking remains largely unresolved.

The ecological interpretation of plant sound responses also requires careful discipline. Recent analyses argue that, although plants emit acoustic signals under stress and can respond to certain vibration regimes, there is still no convincing evidence that plants communicate with one another through a dedicated acoustic channel over ecologically meaningful distances.^68^ Likewise, the renewed discussion of plant-emitted sounds has emphasized that many detected emissions are best understood as consequences of hydraulic events such as xylem cavitation rather than intentional signals.^69^ These clarifications are useful because they do not weaken the field; rather, they sharpen it. They redirect attention from speculative claims about plant “language” toward experimentally tractable questions about mechanoperception, physiological coupling, and the ecological relevance of specific sound classes such as pollinator buzzes, herbivore chewing vibrations, root–zone vibrations, and anthropogenic vibratory noise. Notably, recent work on anthropogenic vibratory noise suggests that chronic environmental vibrations can alter plant growth trajectories, raising the possibility that agricultural and urban soundscapes are already shaping plant performance in ways that remain largely unmeasured.

From an applied standpoint, the future of plant acoustics likely lies along two complementary tracks. The first is intervention: deliberately using sound or vibration to prime seeds, increase propagation efficiency, modulate ripening, support stress resilience, or bias metabolism toward desired agronomic or nutritional outputs. The second is diagnostics: listening to plant-generated acoustic emissions to infer drought status, hydraulic failure, ripening progression, or disease onset in a non-invasive manner. These two tracks should be developed together. If we can understand which mechanical signatures plants emit under stress and which externally applied signatures reliably shift the plant state, it may become possible to build closed-loop cultivation systems in which acoustic sensing guides acoustic intervention. Such systems would be especially attractive in protected cultivation, vertical farming, high-value nurseries, post-harvest handling chains, and precision horticulture, where environmental control is already high and trait-specific optimization is economically realistic. Nevertheless, for broad-acre agriculture, practical deployment will depend on energy cost, scalability, field robustness, and whether acoustic gains exceed those available from simpler agronomic interventions.

Overall, the evidence reviewed in this manuscript justifies cautious optimism. Sound and vibration are not fringe variables in plant biology; they are part of the mechanical environment to which plants are continuously exposed, and under appropriate conditions, they can measurably alter hormonal organization, metabolism, stress physiology, development, and crop-relevant traits. The challenge for the next phase of the field is therefore not to accumulate more isolated demonstrations that “sound affects plants”, but to build a predictive framework that links wave properties to cellular perception, signaling topology, organ-level mechanics, ecological function, and agronomic outcome. Achieving this goal will require more rigorous biophysics, stronger attention to the plant developmental context, integration with root and microbiome biology, and multi-scale experiments that connect molecular events to stable performance in realistic production systems. If those challenges are met, sound may become a credible tool in sustainable crop management, not as a universal cure but as a finely tunable physical input within the broader toolkit of ecological agriculture.
